# Prediction of Antigenic Distance in Influenza A Using Attribute Network Embedding

**DOI:** 10.3390/v15071478

**Published:** 2023-06-29

**Authors:** Fujun Peng, Yuanling Xia, Weihua Li

**Affiliations:** 1 School of Information Science and Engineering, Yunnan University, Kunming 650500, China; john7@mail.ynu.edu.cn; 2 State Key Laboratory for Conservation and Utilization of Bio-Resources in Yunnan, Yunnan University, Kunming 650500, China; xiayl@ynu.edu.cn

**Keywords:** influenza, H3N2, antigenic distance, hemagglutinin, attribute network embedding

## Abstract

Owing to the rapid changes in the antigenicity of influenza viruses, it is difficult for humans to obtain lasting immunity through antiviral therapy. Hence, tracking the dynamic changes in the antigenicity of influenza viruses can provide a basis for vaccines and drug treatments to cope with the spread of influenza viruses. In this paper, we developed a novel quantitative prediction method to predict the antigenic distance between virus strains using attribute network embedding techniques. An antigenic network is built to model and combine the genetic and antigenic characteristics of the influenza A virus H3N2, using the continuous distributed representation of the virus strain protein sequence (ProtVec) as a node attribute and the antigenic distance between virus strains as an edge weight. The results show a strong positive correlation between supplementing genetic features and antigenic distance prediction accuracy. Further analysis indicates that our prediction model can comprehensively and accurately track the differences in antigenic distances between vaccines and influenza virus strains, and it outperforms existing methods in predicting antigenic distances between strains.

## 1. Introduction

Influenza A virus is a negative-sense single-stranded RNA virus, and its viral membrane is mainly composed of two surface glycoproteins: hemagglutinin (HA) and neuraminidase (NA). Existing influenza A viruses are classified into 18 HA subtypes and 11 NA subtypes according to different combinations of HA and NA. Each subtype has distinct pathogenic characteristics and antigenicity. Currently, the H3N2 and H1N1 subtypes are the main seasonal influenza viruses circulating in humans. Among them, the H3N2 virus was first discovered during the 1968 Hong Kong influenza pandemic and has since been continuously circulating globally [1,2]. Seasonal influenza is expected to cause 290,000–640,000 respiratory-related fatalities worldwide yearly [3].

HA is the primary surface protein of influenza A virus and is crucial for the virus’s entry into host cells. The receptor-binding site on the HA protein can bind to sialic acid receptors on host cells, thereby mediating the entry of viral particles into cells and causing infection in humans [4]. Consequently, many vaccines have been developed targeting the receptor-binding site of the HA protein to protect us from future seasonal influenza virus infections [2,5]. The quaternary structure of the HA protein is a homotrimer, with each monomer composed of two subunits, HA1 and HA2. The HA1 subunit contains antigenic determinant regions and critical binding sites for the virus to attach to host cells. Regrettably, mutations in the HA1 subunit can influence the virus’s antigenicity and receptor binding affinity, and the mutation rate is typically higher than in other regions [6]. Antigenic drift caused by accumulated sequence mutations greatly impedes the progress in the development of drug treatments and vaccines against potential influenza viruses. Therefore, the rapid detection of antigenic variation and accurate quantification of antigenic variation are crucial for designing and screening effective vaccines.

Numerous research studies have been conducted using HA protein sequence or structure to generate theoretical models and infer antigenic similarity based on sequence similarity [7,8,9,10,11,12,13,14,15,16,17,18]. Liao et al. [8] used the difference in each non-conserved residue between each pair of HA1 protein sequences as a feature to predict the antigenic distance between HA sequences based on a multiple linear regression model. Ref. [18] used a fitting model to infer two fitness components of the strains that were prevalent in a given year. Then, based on the fitness and frequency of each strain, they predicted the frequency of their descendant strains in the following year. Additionally, the study by Christopher et al. [16] first showed a good correlation between the results of the experimental antigenicity measurements and the antigenic distance prediction based on sequences. Other studies [19,20,21] further predicted the antigenic variations between influenza vaccines and circulating strains by exploring amino acid sequence mutations that identify epitope regions and their association with seasonal influenza. In order to enhance the performance of mutation prediction for particular residue sites of the influenza A virus, Yin et al. [19] built a time series sample by simulating the evolutionary path of HA sequences. Ref. [22] encoded protein sequences into numerical matrices, and subsequently input these matrices into downstream machine learning models, which was shown to improve the accuracy of predicting influenza antigenicity.

The immune efficacy of influenza vaccines mainly depends on how closely the vaccine and circulating virus strains match one another antigenically, so antigenic difference analysis is crucial for selecting vaccine strains [10]. The hemagglutination inhibition (HI) assay has currently established itself as a standard technique for determining the antigenic distance between current circulating influenza virus strains and reference vaccines [23]. The titer acquired from the HI assay is used to generate antigenic cartography that visualizes the antigenic characteristics of different virus strains on a two-dimensional plane. In antigenic maps, we can intuitively observe the antigenic distances and similarities between different viral strains, and then analyze the virus transmission patterns and vaccine strategies accordingly. Smith et al. [24], based on Lapedes and Farber’s [25] metric multidimensional scaling method (MDS), plot antigenic characteristics on the antigenic cartography. The Euclidean distance in antigenic cartography directly describes the antigenic distance, thus providing a reliable quantitative interpretation of antigenic differences.

In order to improve conventional cross-reactivity experiments, Cai et al. [26] believe that modeling to reduce temporal bias in the distribution of HI data is important in antigenic mapping. They used a low-rank matrix completion method to complete the HI titer matrix and then applied the improved MDS method (MC-MDS) to generate the antigenic map. Neher et al. [20] introduced virus potency to explain the systematic changes in HI titers of a virus in multiple sera and used it as the basis for implementing and validating the standardized log-transformed titers relative to the homologous titer. Based on this observation, Lee [27] and Qiu [28] suggested improved antigenic distance calculation methods to reduce the impact of variations in experimental conditions.

Some efforts have been devoted to exploring the relationship between the genetic and antigenic characterization of influenza viruses. According to Koel et al. [29], genetic changes in H3N2 viruses are relatively persistent, while changes in antigenic features recognizable by the human immune system occur intermittently. This suggests that influenza viruses evolve in a way that evades recognition by the human immune system, making the development of effective vaccines more challenging. To conduct a cross-study of genetic and antigenic characterization, ref. [30] mapped the antigenic distance from HI titer data onto the HA lineage. They used available antigenic and genomic sequence data to explain the antigenic novelty and virus transmission rates across the population, and then determined the antigenic changes between clades of high growth. Bedford et al. [31] combined antigenic maps with genetic information on the four human influenza virus subtypes and found that the H3N2 subtype’s antigenic phenotype evolves faster than the other three subtypes. Moreover, there is a strong correlation between the antigenic drift of each influenza strain and the number of new influenza cases each year.

**Figure 1:**
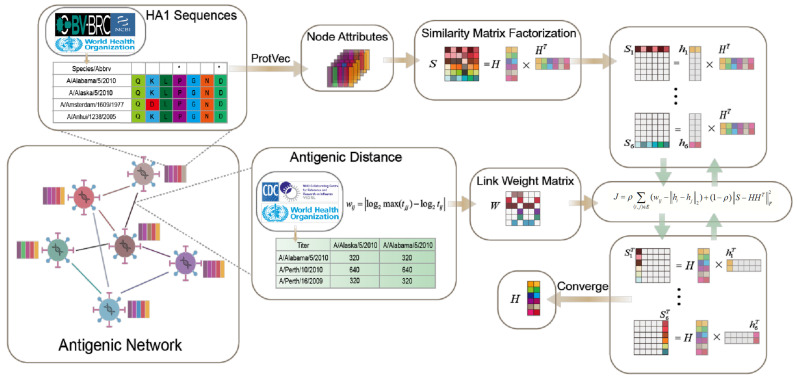
Antigenic network representation learning frame based on AANE.

Previous works have tended to analyze the genetic and antigenic patterns of viruses in isolation. This work aims to use a unified space composed of antigenic and genetic features to model and analyze the evolutionary dynamics of influenza viruses, with the main challenges being how to integrate genetic information represented by amino acid sequences into the space and how to predict antigenic distance quantitatively. To this end, we propose an effective framework to jointly model antigenic and genetic features through antigenic network representation learning with the ProtVec of HA1 sequences as node attributes and antigenic distance converted by HI titer as edge weights. Then, our model learned effective virus representations by introducing node attribute affinity to predict antigenic distances. We applied our proposed model to the H3N2 dataset, performed antigenic distance prediction tasks using the workflow shown in Figure 1, and studied the antigenic evolutionary dynamics of the H3N2 virus. The model takes two inputs: the attribute matrix capturing node attributes, embedded using ProtVec [32], and the link weight matrix representing antigen distances. By calculating the similarity between attribute matrices, it reveals relationships and patterns among node attributes. The link weight matrix quantifies distances between strains using an antigenic distance formula. As the output, the model provides embedding vector representations of strain nodes. These vectors encode the essential characteristics and relationships of strains in a low-dimensional latent space.

Compared with previous methods, we significantly reduced the root-mean-square error of the prediction results and the classification index of antigenic differences. Through vector analysis based on representation learning, we found that the Pearson correlation between genetic distance and antigenic distance (between antigenic clusters) was 0.8694 to 0.9573, while the correlation within antigenic clusters was only 0.7119 to 0.8556. This suggests high global correspondence and some local differences between influenza genetic and antigenic evolution. Eventually, we found that a historical genetic variation of 0.05 ± 0.004813 led to antigenic drift events of H3N2 influenza virus.

## 2. Materials and Methods

### 2.1. Datasets

We used Trevor Bedford’s benchmark dataset [31], which contains 402 H3N2 virus isolates dated from 1968 to 2011.

#### 2.1.1. HI Titers

HI titer data were obtained from official documents published by the World Health Organization. Under the framework of the World Health Organization’s global influenza surveillance network, influenza centers in various countries collect viral isolates of seasonal influenza and use the HI assay to calculate titers for antigenic characterization. The range of HI titers typically spans from 10 to 10,240, as lower dilutions may be subject to potential non-specific inhibition, while higher titers are usually not used. Due to the lack of sensitivity of HI assays beyond a certain threshold, accurate data for experimental results on H3N2 strains mainly exist between strains separated by no more than 14 years. At the same time, some assay data only retain the threshold value, such as “<40”. Specifically, we arrange the antigen and antibody of the HI assay experiments in a matrix according to their chronological order. This results in three types of data: (I) conventional accurate HI titer data showing a band-shaped distribution close to the diagonal of the matrix; (II) data that are lower or higher than a certain threshold, which is typically slightly deviated from the diagonal; and (III) entries lacking experimental data, which are more likely to occur in positions that are significantly off the diagonal.

The HI titer dataset used in our experiments includes 402 viral isolates and 114 antiserum samples. Of these isolates, 187 are from Europe and 215 are from other parts of the world. We obtained a total of 8599 individual HI titer values in the dataset, of which 1110 (12.9%) are type (II) data, i.e., values lower than a certain threshold indicated by “<t”, where t∈{5,10,20,40,80,160}. Considering experimental variations in each study, we removed the top 10% of experimental values for each isolate pair $\left|t_{ij}-\bar{t_{ij}}\right|$ arranged in descending order and calculated the average of the remaining values as the mean titer between isolate *i* and reference isolate *j*.

In actuality, there are three difficulties in interpreting the results of hemagglutination inhibition (HI) assays. Firstly, in order to obtain optimal and reliable results, measurements must be performed under specific experimental conditions such as incubation time, red blood cell concentration, and red blood cell type, which can lead to differences in the results obtained under different experimental conditions. Secondly, HI titers are influenced by the affinity of hemagglutinin for red blood cells, and, to some extent, reflect differences in affinity [33]. In addition, HI assays depend heavily on the antibodies that bind near the receptor-binding domain, and therefore tend to measure responses to specific epitopes. Finally, due to the impossibility of performing HI assays for all pairs of antigens and antisera reactions, the combination of multiple datasets often leads to an incomplete HI titer matrix.

Smith [24] proposed a way of measuring the antigenic distance between two viruses based on HI titer data, where the antigenic distance between strain *i* and *j* is defined as follows:

(1) $$w_{ij}=b_{j}-\log_{2}\left(t_{ij}\right)$$

where $b_{j}$ represents the maximum titer value of the serum *j*, i.e., $b_{j}=\log_{2}\max\left(t_{j}\right)$. When the HI titer value is of the (I) type, *t* represents the maximum dilution of serum *j* that is necessary to inhibit the cell agglutination caused by the virus strain *i*. When the HI titer data are of the “<t” type, $t_{ij}=t$.

To enhance the traditional cross-reactivity assay method and reduce the impact of differences in receptor binding affinities among different virus strains, we were inspired by Neher’s method [20] of calculating relative titers and used the following formula to calculate antigenic distance:

(2) $$w_{ij}=\left|\log_{2}\max\left(t_{jj}\right)-\log_{2}\left(t_{ij}\right)\right|$$

According to the above formula, the antigenic distance between strain *i* and antiserum *j* is converted into the deviation value between the relative titer experimental value of antiserum *j* itself and the titer experimental value of strain *i* relative to antiserum *j*, where $t_{jj}$ and $t_{ij}$ denotes the titer data between strains. This method, as a supplement, can effectively enhance the accuracy and reproducibility of cross-reactivity experiments and has better application effects in analyzing and comparing the receptor binding affinity between different virus strains. For two virus strains with an antigenic distance greater than 4, their antigenicity is considered different, leading to immune escape. Otherwise, they are regarded as antigenically similar. After processing and calculating according to the above method, we finally identified 1543 antigenic difference pairs and 2744 antigenic similarity pairs among 402 strains.

The logarithmic linear correlation concentration ratio defined by the shape space theory of Lapedes and Farber [25] is currently the most widely used method for calculating antigenic distance.

(3) $$w_{ij}=\log\sqrt{\frac{t_{ii}t_{jj}}{t_{ij}t_{ji}}}$$

**Figure 2:**
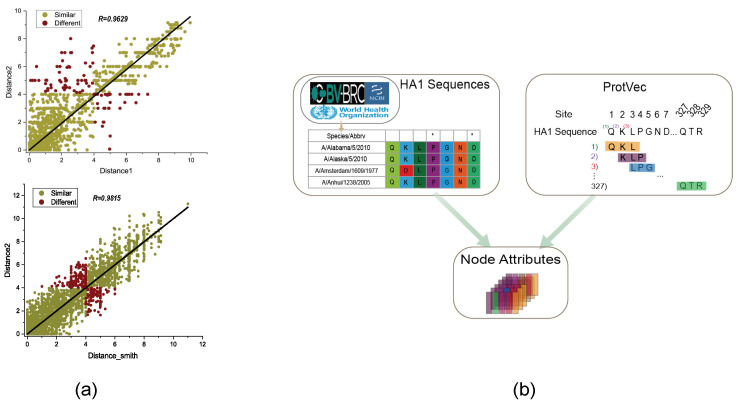
(**a**) The graph illustrates the relationship between the antigenic distance determined by Formula (3) (x-axis) and the proposed calculation method in this paper (y-axis). The green circles represent consistent antigenicity comparisons (i.e., similar or dissimilar) between the two calculation methods, while the red circles represent inconsistent results. The black solid line is the best linear fit with zero intercept, and the correlation between the two antigenic distance calculation methods is 0.9629 with a 95% confidence interval of 94.86% to 97.68%. There is a correlation of 0.9815 between our proposed method and the method used by Smith. (**b**) Using H3N2 HA1 sequence data for ProtVec continuously distributed the representation as node attribute information. To construct the antigenic diversity network and represent the node attribute information, we embedded the HA1 amino acid sequences of 329 in length into a node attribute information matrix using ProtVec. ProtVec applies Skip–Gram to learn the distributed embedding vector representation of influenza virus amino acid sequences, representing continuous triplets of amino acid sequences as 100-dimensional vectors. Through ProtVec representation, each node’s attribute information matrix can be obtained.

There are only 1403 entries for antigenic distance, as calculated by Formula (3). However, trying to make comparable predictions from the sparse and coarser-grained data in these 1403 entries is more difficult. We validated our proposed method’s significant consistency with the popular method by performing a correlation analysis between the standardized logarithmic transformed antigenic distance calculations and the calculation method described in Formula (3) (measured with the Pearson correlation coefficient (PCC), R = 0.9629, 95% confidence interval: 94.86% to 97.68%, see Figure 2a). Similarly, we also calculated a correlation of 0.9815 between our proposed method and the method used by Smith. Based on these findings, it can be concluded that the standardized logarithmic transformation method for antigenic distance calculations can expand the utilization of influenza monitoring data, and we can better predict major antigenic changes.

A number of studies [20,27,34] have made efforts to explore the relationship between point mutations and influenza outbreaks based on a small number of amino acid characteristics. However, these models only measure the contribution of the selected amino acids as individuals. On the one hand, the lack of background of composite effects is due to the fact that amino acid changes in HA form a three-dimensional structure spatially. A previous study [35] analyzed the contributions of individual mutations and their related combined effects through CNN models. In addition, different amino acid residues have a significant impact on the antigenicity of H3N2 virus, and how to measure the contribution of different residues is one of the key issues in sequence processing.

#### 2.1.2. HA Sequences

The HA sequences of 402 virus strains were collected from databases such as NCBI and GISAID. The lengths of HA1 sequences were 329 for influenza A/H3N2. Sequences containing missing or abnormal amino acids (i.e., “-”) were manually and automatically removed, and aligned using Mega. From 1968 to 2011, there were 0 to 30 amino acid changes between these strains. We defined the genetic distance between two HA sequences as the sum of pairwise distances between their 329 amino acids. To construct the antigenic network and represent the attribute information of its nodes, we embedded the HA sequences into a node attribute information matrix based on ProtVec. ProtVec [32] applies Skip–Gram to learn the distributed embedding vector representations of influenza virus amino acid sequences, representing three contiguous amino acid sequences as 100-dimensional vectors. By training only on protein sequences, the ProtVec feature extraction method is able to capture various meaningful physical and chemical properties, and can serve as an informative and dense representation of biological sequences in protein family classification. Specifically, each HA sequence is represented as a list of 327 contiguous 3 g, and a 327 × 100 matrix is generated as the node attribute information in Figure 2b. To represent 3 g sequences containing “-” in our study, we utilize the “<unk>” vector from ProtVec.

### 2.2. Antigenic Network Representation Learning

In recent years, some interpretable attributed network embedding algorithms have emerged. For example, accelerated attributed network embedding (AANE) [36] decomposes the heterogeneous information similarity matrix and penalizes embedding differences between adjacent nodes to preserve the similarity of nodes in the original network space on the new continuous vector representation. Inspired by this observation, this paper discusses the possibility of integrating the node attribute information similarity matrix represented by the HA sequence of influenza virus H3N2 and the topological structure represented by antigenic distance into the network embedding representation learning, and whether this method can help to better learn node vectors in the antigenic network. In addition, the rapid mutation and seasonal epidemics require the scalability of antigenic network embedding representation. AANE embeds noisy network topology and node attributes to improve the model’s time complexity and convergence speed.

The graph-based attribute network embedding method demonstrates strong robustness in handling missing data. In cases where certain antigenic distances are missing, the embedding method can utilize the attribute information of the nodes to impute the missing values, thereby partially compensating for the loss of information. By using the ProtVec representation of the genetic sequences as node attributes, we were able to exploit the informative features of each strain. This not only enriches the available information within the network but also facilitates the extraction of meaningful patterns and relationships between strains. Meanwhile, graph networks inherently possess the ability to model non-linear interactions and dependencies, which may be crucial for capturing the complexity of virus strain evolution. This flexibility allows our model to better capture the intricate patterns and dynamics present in the antigenic distance data.

We define the embedding learning of the antigenic network as follows for an antigenic network G=(V,E,W), where $V=\{v_{1},v_{2},...,v_{n}\}$ represents the set of *n* strain nodes. The attributes of each node are represented by the matrix $p_{i}\in R^{(k-2)\times m}$, which is obtained by embedding the amino acid sequence of length *k* using ProtVec. There is also a set of edges between strain nodes in the network $E={\left\{e_{ij}\right\}}_{i,j=1}^{n}$, and each edge (i,j)∈E is associated with a non-negative antigenic distance $w_{ij}\in W$. $e_{ij}$ is an unobserved edge when $w_{ij}=-1$. This study assumes that the antigenic distances between strains are non-negative and symmetric, used to measure the edge weights between antigenic network nodes. We use AANE for embedding learning to obtain a *d*-dimensional vector $h_{i}\in H$ for nodes $v_{i}$. This embedding method produces a representation of every node in the network space that combines genetic and antigenic properties, allowing *H* to maintain the adjacency of both the topological structure and node attributes simultaneously.

The strain vectors learned through network embedding representation learning should have the following four advantages: (1) low dimensionality, i.e., the embedding dimension *d* should be smaller than the length of the original sequence, in order to improve the efficiency of downstream tasks; (2) preserving the antigenic features of the original antigenic network structure, i.e., nodes with structural similarity should still be similar in the new space; (3) preserving the genetic features of the original antigenic network, i.e., the similarity of original HA1 sequence should be well captured to complement rather than degrade the expression of the network structure; and (4) the pairwise similarity of node embedding vectors should reflect the pairwise similarity of nodes in the original antigenic network. Compared with pure network embedding, network embedding representation *H*, which concurrently maintains the topological structure and node attribute information, performs better on the link weight prediction challenge.

#### 2.2.1. Network Topological Structure Modeling

To maintain the proximity of nodes in the network while improving the performance of antigenic distance prediction, we first assume that strains with similar topological structures or connected by smaller weighted edges are more likely to have similar embedding vector representations. To accomplish this, we suggest the following loss function to reduce the differences in embedding between linked strain nodes:

(4) $$L_{G}=\sum_{(i,j)\in E}(w_{ij}-{∥h_{i}-h_{j}∥}_{2})$$

where $h_{i}$ and $h_{j}$ are the vector representations of node $v_{i}$ and node $v_{j}$, and $w_{ij}$ is the edge weight between them. The key idea of this loss function is to minimize $w_{ij}-{∥h_{i}-h_{j}∥}_{2}$. For the antigenic network, a smaller antigenic distance $w_{ij}$ needs to force the difference between the vector representations $h_{i}$ and $h_{j}$ of two strains to be smaller. By using the 2-norm ${∥h_{i}-h_{j}∥}_{2}$ as the difference metric between vectors, it can not only characterize the distance between vectors but also alleviate the negative effects of outliers and missing data.

#### 2.2.2. Node Attribute Proximity Modeling

According to social science theories such as homophily and social influence [37,38], node attribute information is closely related to network topology. Thus, the similarity of nodes in the network space should be consistent with the similarity of nodes in the attribute space. Inspired by symmetric matrix factorization, the product of *H* and $H^{T}$ approximates the node attribute similarity matrix *S*. The basic idea is to force the dot product of the embedding representation of $h_{i}$ and $h_{j}$ to be similar to the corresponding attribute similarity matrix. Therefore, to maintain node attribute proximity, we define the loss function as follows:

(5) $$L_{S}={∥S-HH^{T}∥}_{F}^{2}=\sum_{i=1}^{n}\sum_{j=1}^{n}{\left(s_{ij}-h_{i}h_{j}^{T}\right)}^{2}$$

where the matrix $S\in R^{n\times n}$ represents the 2-norm between the node attribute matrices, capturing the attribute similarity and the differences between different strains in the joint space. Specifically, given the node attribute information $a,b\in R^{(k-2)\times m}$ based on ProtVec representation, the formula for calculating node attribute similarity is as follows:

(6) $$s_{ij}=\sqrt{\sum_{i}^{k-2}\sum_{j}^{m}{\left(a_{ij}-b_{ij}\right)}^{2}}$$

#### 2.2.3. Antigenic Network Embedding Representation Learning

According to Equations (4) and (5), we have implemented two loss functions, $L_{G}$ and $L_{S}$, to fit the similarity between network topology and node attributes. To make them complement each other and form a unified network representation space, our optimization objective is the following function:

(7) $$J=\rho \sum_{(i,j)\in E}(w_{ij}-{∥h_{i}-h_{j}∥}_{2})+{∥S-HH^{T}∥}_{F}^{2}$$

ρ functions as both a regularization parameter that balances the number of clusters and a global parameter that defines the contribution of network structure and attribute information to the node representation. The intuitive explanation is that when it approaches 0, the network topology cannot affect the final node representation *H*, and each strain will reflect the similarity of node attributes. When ρ is sufficiently large, the vector representation of all nodes will tend to reflect the network’s topology fully. We need to use the Euclidean distance in the new network space to calculate the predicted antigenic distance between any two strains $v_{i}$ and $v_{j}$. To ensure the accuracy and reliability of our predictions, we will follow widely adopted methods for validating network embedding quality.

## 3. Results

We will address the following three questions through experiments: (1) Does supplementing genetic information represented by HA1 sequences better predict antigenic distances based on antigenic network learning compared to using only antigenic features? (2) Can the AANE method used in antigenic network learning achieve more accurate antigenic distance prediction than other attribute network embedding learning methods? We will also further discuss the sensitivity of model parameters, including the regularization parameter ρ of the loss function and the dimension *d* of the node representation vector. (3) By modeling genetic and antigenic features in a combined space, can we gain new insights into the evolution of H3N2 viruses?

### 3.1. Baseline

To answer the aforementioned questions, we will use the following models as baselines and describe them in detail:

- Node2vec [39] is a Skip–Gram-based algorithm that generates node sequences through biased random walks. Hyperparameters *p* and *q* are used to control the random walks, and we adjust them according to the original paper. When *p* and *q* are both 1, node2vec is equivalent to DeepWalk. Node2Vec only uses antigenic distance for an edge-weighted biased random walk.
- LINE [40] constructs the network using only antigenic distance and generates context nodes through breadth-first search, where a node’s neighboring nodes are limited to those that are at most two hops away. LINE models both first-order and second-order similarities for each node and concatenates the two learned embedding vectors according to the actual scenario.
- LINE1 constructs the network using only antigenic distance and models first-order similarity to learn node representation, which mainly constrains directly connected nodes.
- LINE2 also constructs the network using only antigenic distances, but focuses on the neighborhood similarity of nodes and learns node representation by preserving second-order similarity.
- Attri2vec [41] learns node representation by performing linear or non-linear mapping on node attributes. To preserve structural similarity, it uses the DeepWalk learning mechanism so that nodes with similar random walk contexts have similar dense representations in the subspace. This is achieved by maximizing the probability of the appearance of context nodes conditioned on the target representation.
- GCN [42] is a graph-specific model that applies convolution on graph nodes to generate representations for each node.
- By employing the masked self-attention layer, GAT [43] overcomes certain limitations present in existing methods. The key aspect of GAT lies in stacking multiple layers, where each layer can implicitly assign varying weights to neighboring nodes without the need for costly matrix operations or prior knowledge of the graph structure.
- GraphSAGE [44] utilizes local neighborhood sampling to aggregate features and generate embeddings. Subsequently, a minibatch forward propagation algorithm is employed to train the data.
- GALA [45] proposes a symmetric graph convolutional autoencoder for generating low-dimensional latent representations of graphs. Compared to existing graph autoencoders, our model features a newly designed symmetric decoder that effectively utilizes the graph structure for reconstructing node features.
- TADW [46] not only considers the structural information of nodes but also utilizes the text information of nodes. It implements the DeepWalk idea through matrix factorization and introduces node text information to improve the expression of embedding vectors.

### 3.2. Evaluation of Antigenic Distance Prediction Performance

The performance of antigenic distance prediction is evaluated using two metrics: the root mean square error (RMSE) and the Pearson correlation coefficient (PCC) between the predicted and actual antigenic distances. These metrics reflect different aspects of prediction performance: RMSE amplifies the differences between larger errors and quantifies the degree of proximity between the prediction and the average true value, while PCC measures the relative trend between the two. Given *n* true antigenic distances $w_{ij}$ and predicted antigenic distances $d_{ij}$, the corresponding metrics are defined as follows:

(8) $$RMSE=\sqrt{\frac{1}{n}\sum_{(i,j)\in E}{\left(w_{ij}-d_{ij}\right)}^{2}}$$

(9) $$PCC=\frac{\sum_{(i,j)\in E}\left(w_{ij}-\bar{w_{ij}}\right)\left(d_{ij}-\bar{d_{ij}}\right)}{\sqrt{\sum_{(i,j)\in E}{\left(w_{ij}-\bar{w_{ij}}\right)}^{2}\sqrt{\sum_{(i,j)\in E}{\left(d_{ij}-\bar{d_{ij}}\right)}^{2}}}}$$

Furthermore, we will evaluate the model’s ability to detect antigenic drift based on the prediction results. We will assess this capability using four evaluation metrics: accuracy, precision, recall, and F1 score.

**Figure 3:**
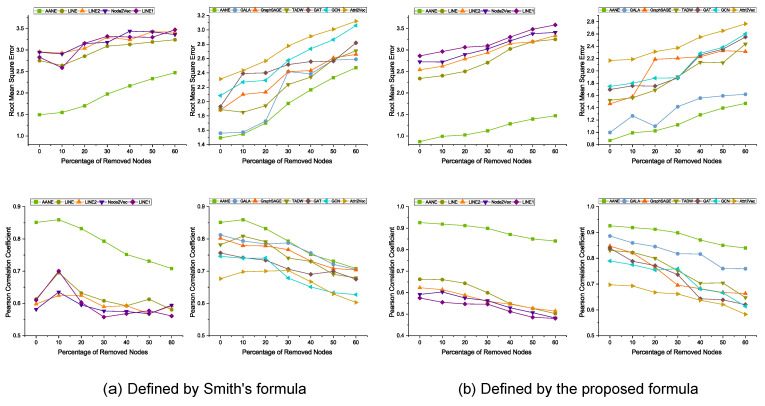
Figure depicts the performance of the antigenic network embedding learning model based on AANE in terms of the RMSE (top) and PCC (bottom) metrics for antigenic distance prediction tasks, using the H3N2 antigenic network dataset (1968–2011) with *d* = 50. This model outperforms all other models. The x-axis represents the percentage of randomly removed nodes from the network (from 0% to 60%), and the y-axis represents the corresponding evaluation metrics. In (**a**) and (**b**), the predicted results of antigenic distance calculation are shown using the formula defined by Smith and the proposed normalized logarithmic transformation formula in this paper, respectively. In the left subplots of (**a**) and (**b**), the models that utilize antigenic distance as the only link weight for antigenic distance prediction are compared with AANE (green line) in terms of RMSE and PCC results. In the right subplots of (**a**) and (**b**), the models that utilize antigenic distance as the link weight and the ProtVec matrix encoding HA as the node attribute for network embedding learning are compared with AANE in terms of RMSE and PCC results (green line).

With a default embedding dimension, *d*, of 50, we set the baseline method’s settings as recommended in the original paper. All experimental results are arithmetic averages of 10 tests. We divide the dataset into a training set and a test set (75% and 25%), and perform multiple rounds of training and evaluation through 3-fold cross-validation by dividing the training set into three subsets and using these subsets alternately as validation sets. Based on the preliminary findings from Figure 3, we found that the methods based on network embedding representation learning achieved significantly better performance than LINE1 in predicting antigenic distances on the H3N2 dataset. We attribute this phenomenon to the following two reasons: (1) the LINE model was applied to millions of data points, which is vastly different from the sample size used in this experiment; and (2) in contrast, LINE1 only models first-order proximity, which cannot capture enough information for link weight prediction tasks.

Next, we then evaluate the impact of merging node attribute information. In order to make a fair comparison with models that utilize node attributes, we use Node2Vec, LINE1, LINE2, and LINE as controls, which only consider antigenic distance as the link weight for node vector learning. These methods learn node vectors only through network structure and then predict antigenic distance. As shown in Figure 3a,b, on the dataset of all 402 nodes, the model based on attribute network representation learning outperforms in terms of RMSE and PCC. This confirms our hypothesis that the combination of genetic and antigenic features proposed by us contributes to antigenic distance prediction tasks. Horizontally, Figure 3a,b suggests that utilizing both network structure and node attribute information is beneficial for downstream link prediction tasks.

To further evaluate how much it improves the performance of antigenic distance prediction in the antigenic network, as well as to verify the robustness of the AANE model, we further reduced the number of nodes in the network by 10%, 20%, 30%, 40%, 50%, and 60%. Each experiment was repeated 5 times and the average was taken to test the model’s robustness in predicting missing antigenic distances. For example, as the number of deleted nodes increased, LINE (grayish green line in Figure 3b) and Node2Vec (bluish violet line in Figure 3b) showed an overall decreasing trend in RMSE, with a final decrease in the predictive performance of 24.6% and 24.9%, respectively.

According to the experimental results in Figure 3a,b, AANE consistently outperformed Attri2Vec, GCN, GAT, GraphSAGE, GALA, and TADW. All of these approaches execute node embedding learning and represent the network using node attributes and link weights. This illustrates how effective AANE is. For example, on the dataset consisting of all nodes, AANE achieved a 38.1% and 13.1% improvement over TADW and GALA, respectively. Although TADW is effective in learning information-based node embeddings using rich node text features, its mechanism is not as straightforward, i.e., a clear objective is not provided for how the network structure and node attributes interact with each other.

**Figure 4:**
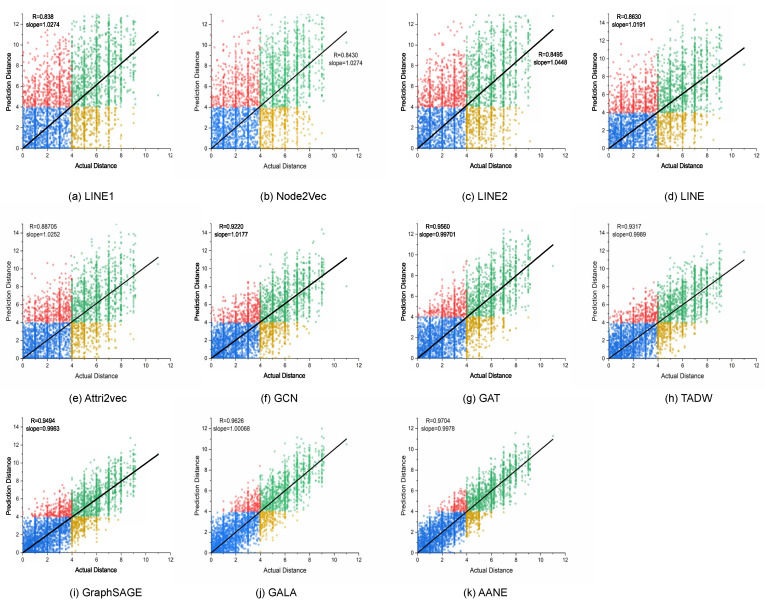
Linear regression analysis of predicted values (y-axis) versus actual values (x-axis) for different models (solid black line). Green dots represent true-positive (TP) predictions; blue dots represent true-negative (TN) predictions; red dots represent false-positive (FP) predictions and yellow dots represent false-negative (FN) predictions.

We compared the predicted antigenic distances of all models with the actual antigenic distances by linear fitting (Figure 4). The results show that the attribute network embedding method had a greater advantage in reducing FN data and increasing TN data. Meanwhile, AANE’s predicted values had good robustness and a more uniform distribution when linearly fitted with the actual distances. The reason for Node2vec’s performance exceeding our expectations may be explained here. We believe that Node2vec does not reflect structural information well. Due to the limited sample size and walk length, it is almost impossible to include two structurally similar nodes in the same sequence through biased walks when the distance between them is very large. This is also related to hyperparameter selection. We set *p* as 1 and *q* as 2. The larger *q* is, the more the embedding tends to express homogeneity. As the number of nodes decreases, nodes with experimental data are often those that are close to their own antigenic distances. Therefore, when expressing network structure, it tends to embed peripheral and central nodes as similar vectors (TN- and FN-predicted values in Figure 4b,f occupy a considerable part). In fact, these predicted values, which fit the original data distribution more closely, can obtain better predictive ability. We followed the author’s recommendation and did not stack higher layers of GCN. Perhaps there will be better performance with more than three layers of GCN, but this is not within the scope of this paper. GCN has the same drawback as Attri2Vec—the over-smoothing problem (that is, after multiple layers of stacking, the node’s representation vectors tend to be consistent, and the nodes are difficult to distinguish). Due to the low-pass filter effect of GCN, the aggregated features continuously merge the node features, which tend to be the same after multiple iterations. GAT has more parameters than GCN and is trained in a full-batch manner. It only considers 1-hop neighbors and does not utilize higher-order neighbors. When higher-order neighbors are used, excessive smoothing is prone to occur. Attri2Vec has a strong bias toward node attributes, so it is not practical to maximize the attribute information difference among strains with no more than 30 different amino acids even after ProtVec representation.

**Figure 5:**
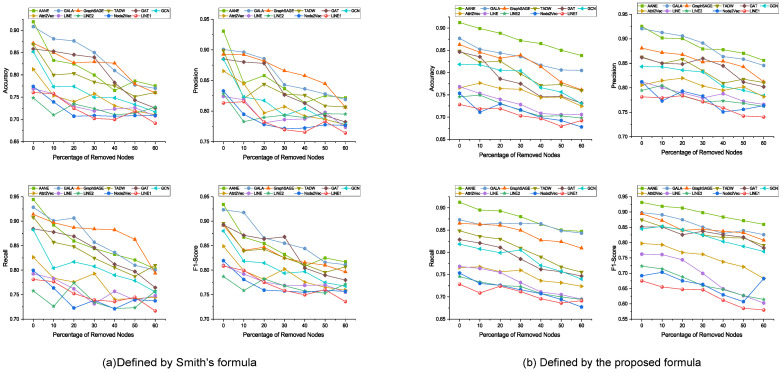
The antigenic distances predicted by the model were converted to antigenic differences (using $D_{\left(i,i+1\right)}=4$ as the threshold for binary classification) and measured on the H3N2 dataset with different classification metrics: accuracy, precision, recall, and F1 score. (**a**) and (**b**) represent the results of predictions using the antigenic distance data calculated using the formula defined by Smith and the antigenic distance data calculated using Equation (3), respectively.

We tested the ability of all models to successfully predict antigenic escape (as described in Section 2.1, where the antigenic distance between two strains is higher than the antigenic escape threshold ($d_{ij}$ = 4)). The main evaluation metrics for qualitative results are accuracy, precision, recall, and F1 score, as shown in Figure 5. Our results show that the AANE model accurately predicted the antigenic distance between two strains with an accuracy of 91.25%, and other metrics also showed significant advantages.

As shown in Figure 3 and Figure 5, we will present a comprehensive analysis including quantitative evaluation and statistical measures to validate the accuracy and reliability of our defined distances. We compared the predictive performance of the antigenic distances obtained with the computational methods defined by Smith on all benchmark models. As for Equation (3), we chose to discard this set of comparisons because of the small number of entries obtained. We could not obtain more substantial results with these network embedding methods. We found erratic metric fluctuations in the sequential reduction in the number of nodes on the antigenic dataset defined by Smith, a phenomenon that occurs in most of the benchmark methods. As we feared, the formula defined by Smith tended to obtain certain fixed values during the calculations performed on the titer data we collected (also found in Figure 2a), even though it allowed us to obtain more entries of antigenic data. This comparative analysis will help to confirm the validity of our proposed method and to obtain a clearer picture of the differences between the “actual” antigenic distances in our study and the established criteria.

### 3.3. Parameter Sensitivity Study

In this section, we looked into the effects of two significant parameters, ρ and *d*. As described in Section 2.2.3, ρ in AANE balances the contribution of network structure and node attributes. To study the effect of ρ, we changed it from $10^{-3}$ to $10^{6}$. As there are up to 35 or more different combinations of hyperparameters (a,b), we only give the optimal value of another parameter *b* under a specific parameter *a*. Table 1 shows the RMSE and PCC results of antigenic distance prediction under different ρ values. Setting $\rho =10^{-3}$ almost ignores the influence of network structure information, and nodes tend to have the same embedding vector representation. As ρ increases, AANE predicts the antigenic distance based on the topological structure, and the performance gradually improves. As shown in the figure, when ρ is close to $10^{5}$, the performance of antigenic distance prediction peaks. When ρ continues to increase, the performance will decrease, as larger ρ values tend to make all nodes too dependent on sparse structural information. However, we cannot directly infer from this so-called optimal value that genetic information only contributes 0.001% to the distance prediction task because the two dimensions are intuitively very different. Nevertheless, from the overall trend of change, it indicates to some extent that genetic information contributes to the advantage of using attribute network embedding for antigenic distance prediction, as shown by the improvement in the RMSE value.

Following the rules we established when building the network representation model (Section 2.2), dimension *d* should be less than 329. Specifically, we changed the embedding dimension from 20 to 150, and Table 1 shows the prediction performance on the dataset. From the results, we found that by increasing *d*, the performance of the method first increases and then remains stable. This indicates that low-dimensional representations perform well in capturing most of the meaningful information. In reality, determining an appropriate dimension is not easy, especially for antigenic distance prediction. Although a lower dimension has lower time and space complexity, it will undoubtedly lose a lot of information originally present in the network. Higher dimensions may improve reconstruction accuracy to some extent, but at the same time, the Euclidean distance between vectors of higher dimensions will likely become larger. Based on our experimental results, we can conclude that model performance is relatively stable within a small range of node embedding dimensions, and performance declines when the node embedding dimension is too small or too large.

### 3.4. Antigenic Evolution Dynamic Analysis

**Figure 6:**
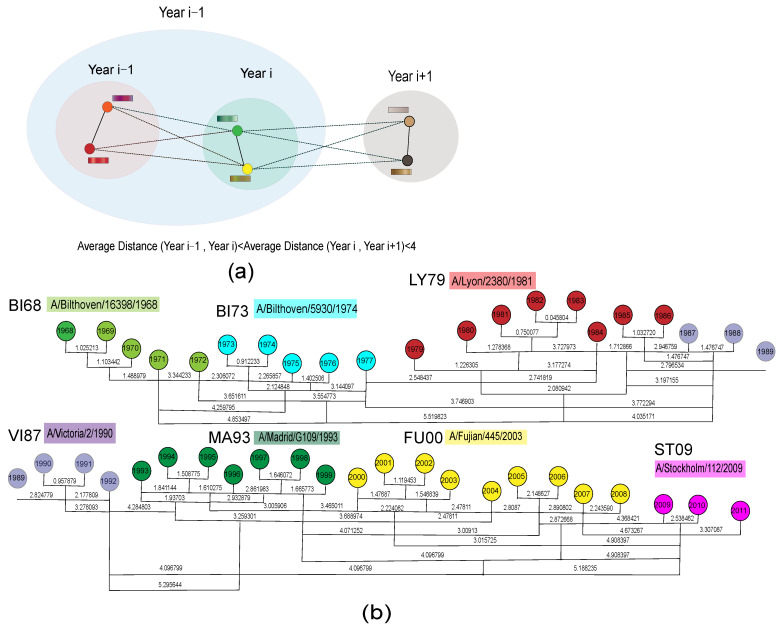
Antigenic clustering over the past four decades (1968–2011). (**a**) During the entire clustering process, adjacent clusters with the smallest antigenic distance in the current collection of all clusters are selected successively and merged into a new cluster without antigenic variation ($D_{\left(i,i+1\right)}<4$). (**b**) Each circle represents all strains in a given year, and the numerical values between two circles represent the average antigenic distance between clusters during the updating process. Adjacent clusters with similar antigenicity are merged into new clusters, and the strain with the smallest antigenic variation in each cluster is used to name the final cluster.

The effectiveness of the model has enabled us to explore the dynamics of influenza antigenic evolution based on vector representation in the joint space. As shown in Figure 6, we first performed preliminary clustering by year and calculated the average antigenic distance $D_{\left(i,i+1\right)}$ between all pairs of strains from year *i* and i+1 for the 44-year period, resulting in 41 data points (except for 1978 in the H3N2 dataset). Then, we merged adjacent clusters that had the smallest antigenic distance ($D_{\left(i,i+1\right)}<4$) without antigenic variation, and each new cluster was named after the earliest year in the cluster. For example, 1982 and 1983 strains had the smallest antigenic distance (0.045804) and were merged into a new cluster, followed by recalculating the average antigenic distance to 1981 and 1984 strains. All strains were finally grouped into seven significant antigenic drift episodes, as seen in Figure 6b. We calculated the antigenic distance between each pair of strains in each event. In an antigenic drift event *E* including *n* strains, the antigenic variation level of strain *i* was defined as

(10) $$C_{i}=\frac{\sum_{j\in E}d_{ij}}{n-1}$$

where $d_{ij}$ represents the antigenic distance between strain *i* and all other strains *j* within the same event *E*. The strain with the smallest antigenic variation value within the current cluster is chosen as the dominant strain (which has the smallest average antigenic distance to all other strains in the cluster) and is used to name the event. Between 1968 and 2011, we discovered seven significant antigenic drift events: BI68, BI73, LY79, VI87, MA93, FU00, and ST09.

**Figure 7:**
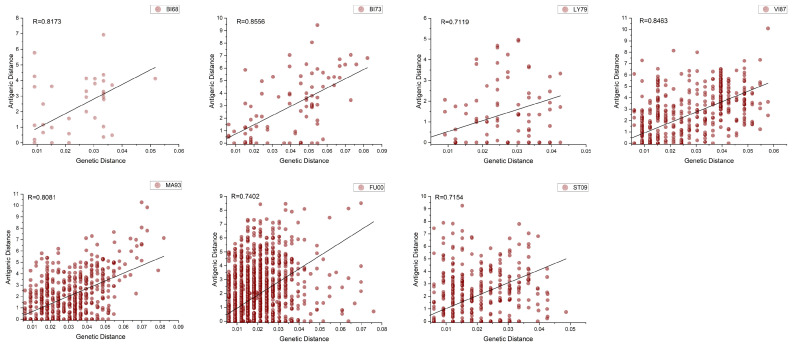
Comparison of the relationship between genetic distance and antigenic distance in the same cluster.

**Figure 8:**
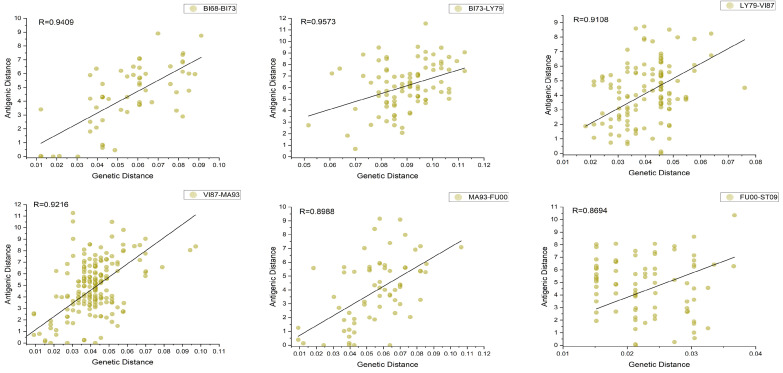
Comparison of the relationship between genetic distance and antigenic distance in adjacent clusters.

Based on the clustering results, we quantified the relationship between antigenic and genetic distances among strains. The genetic distance was calculated from the amino acid sequence differences between strains. In our study, the differences in the number of identified antigenic drift events compared to the research conducted by Smith et al. [24] could be attributed to various factors, including the datasets used, the definition of antigenic distance, and the specific criteria employed to define antigenic drift events. Furthermore, we looked at the roughly evolutionary relationship between H3N2’s genetic and antigenic characteristics. First, for the global time scale, the Pearson correlation coefficient between genetic and antigenic distances was 0.8559, indicating a roughly linear relationship between genetic and antigenic differences during the inter-epochal evolution of influenza, which is consistent with the relationship observed by Smith et al. [24]. We randomly selected some strains within and between each cluster event to calculate their average genetic and antigenic distances. Surprisingly, the genetic–antigenic evolutionary relationship between clusters showed a stronger linear pattern than that within clusters (Figure 7). Moreover, the genetic and antigenic evolution between the seven adjacent antigenic drift events showed a linear correlation of 0.8694 to 0.9573, while the evolution within clusters was characterized by discontinuous development (see Figure 8). Furthermore, we calculated that an average of 0.05 ± 0.004813 units of genetic variation led to the occurrence of an antigenic drift event. However, the distribution of genetic distances between different antigenic drift events varied greatly, and the distribution within clusters was more concentrated, even though they were usually very small. This aligns with earlier studies [47,48], which suggested that strong selection and neutral antigenic evolution alternated during antigenic drift events. As a result, the new vector space learned by the antigenic network representation learning method can explain the short-term and long-term patterns of the relationship between genetic and antigenic distances. Moreover, this method greatly improves the resolution and accuracy of antigenic differences.

## 4. Discussion

Certainly, there have been some notable achievements in the field. For instance, ref. [49] proposed a novel approximation method for antigenic distance, which is based on deep learning in the feature space induced by hemagglutinin protein sequences and convolutional neural networks (CNNs). On the other hand, ref. [50] evaluated the predictive capability of their method by conducting laboratory measurements. We recognize the importance of a thorough comparison and evaluation with other relevant methods. However, the experimental results indicate that our study is an initial exploration of the proposed method and provides a foundation for future investigations and advances.

Historical experience shows that antigenic data cannot significantly reflect genetic data. This article proposes a method based on the AANE model for network representation learning to integrate HA sequence data into the antigenic network structure. This method can quantitatively predict the antigenic differences between strains well. Since the HA sequence and titer data provide different sources of information, it is crucial to capture their key features to learn the comprehensive representation of strains in the antigenic network. For instance, specific epitope positions on the HA protein and the amino acids that make up the epitope may differ due to host species and genotype. The affinity matrix can map specific amino acid mutations between different nodes to the perspective of the entire sequence, which is another reason why we think it should be introduced. Its biological characterization distinguishes the differences in the three continuous amino acid sequences.

In this paper, the antigenic distance is inferred using both genetic and antigenic data, which differs from the positions inferred solely from the HI data. If the HI data are abundant, we expect smaller differences in prediction when genetic data are included (possibly only for H3N2). In contrast, if the HI data are limited, we expect genetic data to play a greater role in determining antigenic positions. As described in Section 3.3, genetic and antigenic features are not complementary, but rather genetic features complement antigenic features to explain quantitative differences in antigenicity. This is in line with our original intention of incorporating genetic information into the network structure to improve antigenic distance prediction performance. Furthermore, our validation of the contribution of hyperparameters of the loss function to the prediction task also supports the same viewpoint.

Little is known about the relationship between influenza virus antigenic phenotype changes and genetic sequence changes. A helpful framework for investigating influenza is provided by the work of Bedford et al. [31], which may be used to identify which alterations to virus genes lead to changes in antigenicity. Our model for antigenic network learning based on AANE optimizes the observed different patterns (such as homogeneity and structure) in the network, proposes a robust objective function, and makes some assumptions about the relationship between the underlying network structure and the prediction task. We can determine its effectiveness and scalability by observing how the learned vectors reflect the relationship between genetic evolution and antigenic evolution. Information about the virus’s “antigenic dynamics” can be reflected in the evolutionary perspective captured between antigenic drift events, especially in the long-term and short-term patterns of antigenic evolution.

## 5. Conclusions

The main contributions of this paper can be summarized as follows. We propose an effective attribute network embedding framework to learn low-dimensional representations of strains from both the node attribute affinity matrix and topological structure information. By expanding the utilization of influenza surveillance data and the representation of sequence biology significance, we validated the basis of joint spatial modeling, which supports the combination of genetic and antigenic features on real datasets. Through the learned low-dimensional representations, we can better predict the antigenic distance between any strains in the network and explore the new dynamics of antigenic evolution.

## Figures and Tables

**Table 1:** Different combinations of the regularization parameter ρ and embedding dimension *d* affect the prediction results of antigenic distance.

Parameters	RMSE	PCC
($\rho =10^{-3}$, d=150) *	2.1994	0.6956
($\rho =10^{-2}$, d=150)	2.0167	0.7354
($\rho =10^{-1}$, d=150)	1.9503	0.7462
(ρ=1, d=150)	1.8559	0.7662
(ρ=10, d=120 )	1.7025	0.7897
($\rho =10^{2}$, d=120)	1.6603	0.8018
($\rho =10^{3}$, d=120)	1.2973	0.8660
($\rho =10^{4}$, d=100)	0.8899	0.9336
($\rho =10^{5}$, d=50)	0.8678	0.9373
($\rho =10^{6}$, d=20)	1.5160	0.8311
(d=20, $\rho =10^{6}$)	1.6102	0.8086
(d=32, $\rho =10^{6}$)	0.9746	0.9197
(d=50, $\rho =10^{5}$)	0.8730	0.9362
(d=64, $\rho =10^{5}$)	0.9120	0.9303
(d=80, $\rho =10^{3}$)	1.1248	0.8965
(d=100, $\rho =10^{4}$)	0.8950	0.9326
(d=120, $\rho =10^{3}$)	1.3108	0.8652
(d=150, $\rho =10^{2}$)	1.7906	0.7753

* The first parameter represents the determined parameter, and the second parameter represents the optimal value under the condition of the first parameter.

## Data Availability

The antigenic dataset of HI titers was compiled by Bedford et al. and can be obtained at https://datadryad.org/stash/dataset/doi:10.5061/dryad.rc515 (accessed on 28 April 2023); the code implementation and documents related to this paper can be obtained at https://github.com/john-darwin/Antigenic-Distance-Prediction (accessed on 29 June 2023).
